# Multiple congenital oral granular cell tumours in a newborn black female: a case report

**DOI:** 10.1186/1757-1626-1-13

**Published:** 2008-05-30

**Authors:** Liviu Feller, Neil H Wood, Avin S Singh, Erich J Raubenheimer, Robin Meyerov, Johan Lemmer

**Affiliations:** 1 Department of Periodontology and Oral Medicine, School of Dentistry, Medunsa Campus, University of Limpopo, Pretoria, South Africa; 2 Department of Maxillofacial and Oral Surgery, School of Dentistry, Medunsa Campus, University of Limpopo, Pretoria, South Africa; 3 Department of Oral Pathology, School of Dentistry, Medunsa Campus, University of Limpopo, Pretoria, South Africa; 4 Department of Periodontology and Oral Medicine, School of Dentistry, Medunsa Campus, University of Limpopo, Pretoria, South Africa; 5 Professor Emeritus: Department of Oral Medicine and Periodontology, School of Dentistry, University of the Witwatersrand, Johannesburg, South Africa

## Abstract

**Introduction:**

Congenital oral granular cell tumour of the newborn is an uncommon benign tumour of uncertain origin. The typical clinical appearance is of a single nodule occurring on the anterior maxillary ridge. In 10% of cases there are multiple lesions. The occurrence of congenital epulis in non-Caucasians is rare.

**Case presentation:**

Two firm pedunculated nodular lesions were noticed in the mouth of a 3-day-old black female: one on the anterior maxillary ridge and the other further posteriorly in the midline of the palate. Both lesions were excised when the baby was nine days old. Microscopic examination of the lesions showed densely packed round to oval cells with abundant granular eosinophilic cytoplasm and uniform nuclei. The diagnosis was congenital granular cell tumour.

**Conclusion:**

Congenital oral granular cell tumour occurs almost exclusively in Caucasian newborns but also rarely in black infants. The parents should be assured of the benign nature and the simple treatment of the condition.

## Introduction

Congenital epulis is an uncommon benign gingival tumour of the newborn. Although the condition has been well reported in the literature, to our knowledge this is the first case to be reported in a black South African infant.

Epulis is a non-specific term connoting a tumour-like mass of the gingiva and the term is used in association with several different gingival lesions, regardless of their aetiology or histogenesis [1]. Since a lesion identical to congenital epulis may occur on the tongue, the term congenital epulis should be discontinued and the term congenital granular cell tumour be used instead [2,3].

## Case presentation and discussion

Three days after the birth of a black female, two lumps were noticed in her mouth. No other oral abnormalities were noted. The lumps did not interfere with feeding or breathing. The mother was HIV-seropositive and had been receiving Neveripine since before the pregnancy. The mother would not allow the HIV-serostatus of the baby to be investigated.

**Figure 1:**
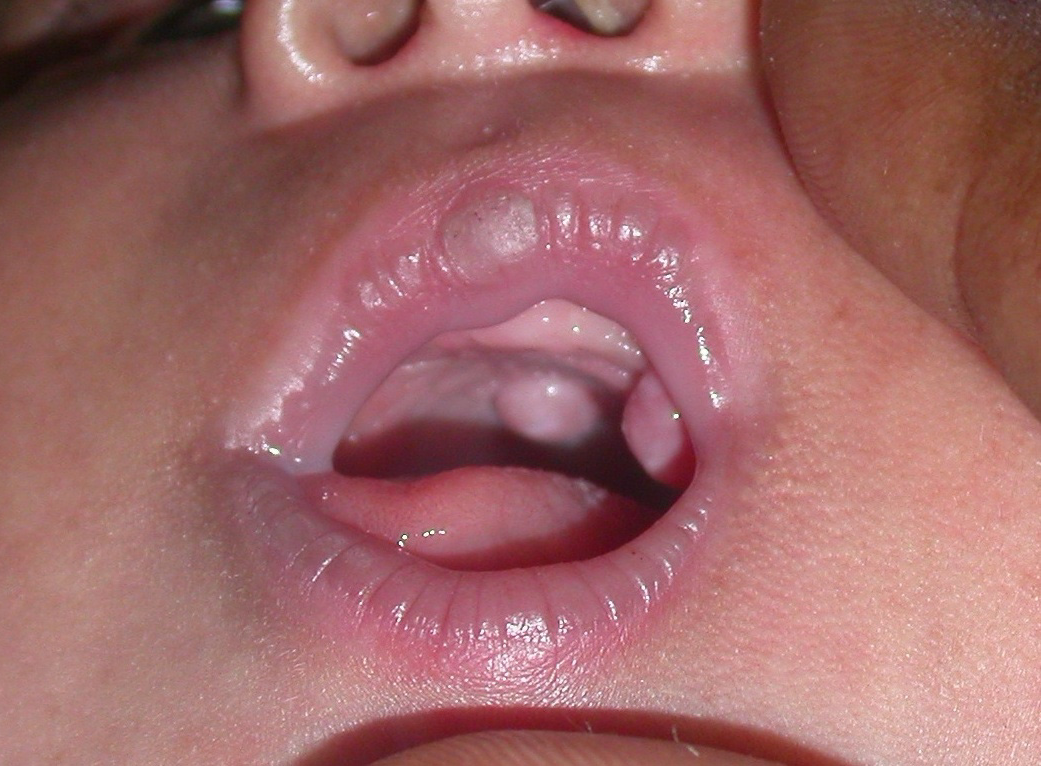
Intraoral photograph showing two lesions: one on the anterior maxillary ridge and another on the middle of the palate.

On examination there were two firm pedunculated nodular lesions (Fig 1), one on the anterior maxillary ridge, 7.5 × 5 mm in size, and the other further posteriorly in the middle of the palate, 6.5 × 5 mm in size (Fig's 2A and 2B).

**Figure 2:**
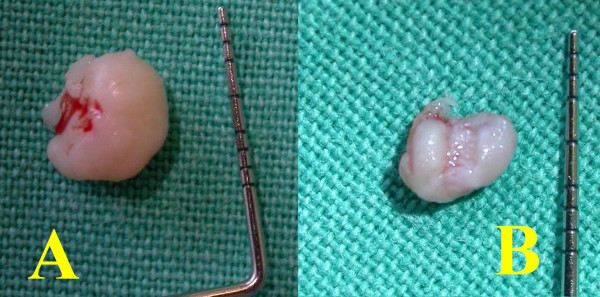
Both lesions were firm, pedunculated and encapsulated.

The differential diagnosis included congenital granular cell tumour (CGCT), odontogenic tumour, teratoma, neuroectodermal tumour, haemangioma and fibroma with a provisional diagnosis of CGCT. Both lesions were excised under general anaesthesia when the baby was 9 days old. Post-operative healing was uneventful.

**Figure 3:**
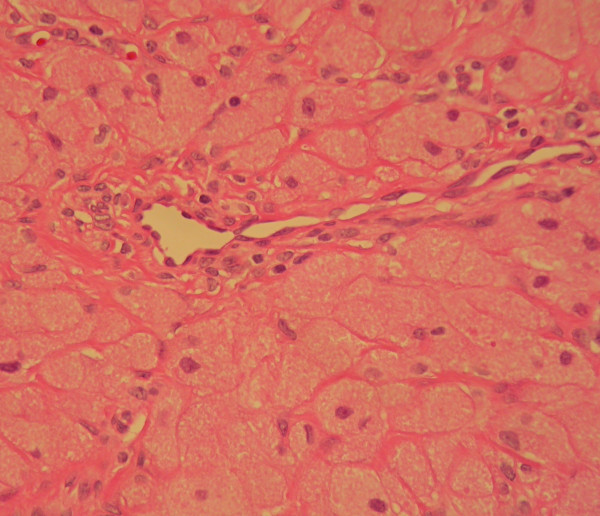
Densely packed round to oval cells with abundant granular eosinophilic cytoplasm and uniform nuclei. Note the thin walled blood vessel in the centre of the field (H&E stain ×200).

Microscopic examination of the lesions showed round or ovoid cells with granular eosinophilic cytoplasm and centrally located nuclei (Fig 3). The masses were covered with a thin stratified squamous epithelium. The definitive diagnosis was CGCT.

CGCT is uncommon and occurs almost exclusively in Caucasian newborns [1]. It is a well defined pedunculated nodule or mass, with a smooth or lobulated surface and is most frequently located on the masticatory mucosa of the maxillary alveolar process. The male to female ratio is 1:8 [4,5]. CGCT can vary in size from several millimeters to several centimeters. Only 10% of subjects with CGCT present with multiple lesions that may affect one or both jaws [2,3]. The condition is not associated with any other congenital abnormalities. Large lesions may interfere with breathing and with feeding [5]. Other than the gingiva, the tongue is the only site rarely affected by CGCT [2,3].

CGCT occurs only in the newborn and is a distinct pathological entity [6]. It may be identified by prenatal sonography, but the findings are not specific and the ultrasonic differential diagnosis would include congenital malformations and various benign or malignant tumours [4].

Local excision is curative. If left untreated malignant transformation never occurs. Spontaneous regression of untreated lesions has been reported [1,3,5,7]. CGCT appears to be more hamartomatous than neoplastic [1,2].

The histogenesis of CGCT is obscure. Its possible origin may be odontogenic, fibroblastic, histiocytic, myogenic or neurogenic cells [1-5,7], or perhaps pluripotential precursors of these cells. The reason for the development only on the alveolar masticatory mucosa or on the tongue remains unexplained [2,3,6-8].

Microscopically, CGCT typically consists of large closely packed polygonal cells with clear granular eosinophilic cytoplasm and centrally positioned nuclei, within a vascular fibrous connective tissue stroma, covered with squamous cell epithelium [1-10]. The granular cells stain positively for vimentin and negatively for S100-protein, oestrogen and progesterone receptors [5,6]. Electron microscopic examination of the granular cells of CGCT shows heterogeneous electron-dense granules, lysosomes, and cytoplasmic lipid droplets [6]. CGCT and granular cell tumour found only in older people have similar microscopic features. However, the latter is less vascular and is typically covered by hyperplastic squamous cell epithelium [1,4].

## Conclusion

CGCT is relatively common in Caucasia newborns but appears to be much less common in black newborns, and the occurrence of more than one lesion in a black infant is rare. The family of an infant with CGCT should be assured of the benign nature and the simple treatment of the condition.

## Consent

Written informed consent was obtained from the parent of the patient for the publication of this case report and for the use of images. A copy of the written consent is available for the Editor-in-Chief of this journal.

## Competing interests

The authors declare that they have no competing interests.

## Authors' contributions

LF, NHW, EJR, RM and JL provided the study concept, ASS and NHW performed the clinical work and case management, EJR performed histopathological studies, LF, NHW and ASS acquired data and performed the data analysis, LF, RM and EJR were responsible for manuscript editing, LF, NHW, ASS, RM and JL reviewed the manuscript. All authors read and approved the final manuscript.
